# Study on Crack Behavior of GH3230 Superalloy Fabricated via High-Throughput Additive Manufacturing

**DOI:** 10.3390/ma17174225

**Published:** 2024-08-27

**Authors:** Xiaoqun Li, Yaqing Hou, Weidong Cai, Hongyao Yu, Xuandong Wang, Fafa Li, Yazhou He, Dupeng He, Hao Zhang

**Affiliations:** 1 China Iron and Steel Research Institute Group, Beijing 100081, China; lixiaoqunwork@163.com (X.L.);; 2 Beijing CISRI-GAONA Materials & Technology Co., Ltd., Beijing 100081, China; 3 ADRAYN Technology Co., Ltd., Chongqing 404100, China

**Keywords:** additive manufacturing, nickel-based superalloys, high-throughput, thermodynamic calculation, deep learning, crack behavior

## Abstract

This study utilized Fe, Co, Ni elemental powders alongside GH3230 pre-alloyed powder as raw materials, employing high-throughput additive manufacturing based on laser powder bed fusion in situ to alloying technology to fabricate the bulk samples library for GH3230 superalloy efficiently. A quantitative identification algorithm for detecting crack and hole defects in additive manufacturing samples was developed. The primary focus was to analyze the composition variations in specimens at varying Fe, Co, and Ni elemental compositions and their impact on crack formation. Experimental results demonstrated that increased laser power improved element distribution uniformity but it proved to be not significantly effective in reducing crack defects. Moreover, augmented Fe and Co alloying content could not eliminate these defects. However, elevated Ni content led to a decrease in the alloy’s solidification cracking index and carbide reduction in solidification products. Notably, a significant reduction in cracks was observed when the Ni content of the alloy reached 63 wt.%, and these defects were nearly eliminated at 67 wt.% Ni content.

## 1. Introduction

Nickel-based superalloy comprises nickel as the base element and doped with up to 10 or more alloying elements [1], primarily used for power generation in the hot sections of aerospace and industrial gas turbines [2] and constituting over 50% of materials in the hot sections of both military and civilian aircraft gas turbine engines [3]. Due to their outstanding high-temperature strength, oxidation resistance, heat corrosion resistance, and resistance to sulfur-based corrosion, Nickel-based superalloy is appropriately suited for use in harsh environments [4]. With the increasing operating temperatures and pressures of engines, the internal structural parts face more stringent, complex, highly integrated, and lightweight requirements [5]. Additionally, nickel-based superalloy contains significant amounts of strengthening elements such as Nb, Mo, or Ti. Controlling the content of these elements adds complexity to processes such as smelting and processing. Furthermore, due to the increasing of a higher thrust-to-weight ratio in aviation engines, combustion chamber temperatures are exceptionally high, which necessitates the design of numerous cooling channels within turbine blades. As a result, structural parts become increasingly complex, and with conventional manufacturing processes, it is hard to meet these requirements [6,7].

Additive manufacturing (AM) is a suitable manufacturing technology; it selectively accumulates single or multiple materials track-by-track or layer-by-layer directly from digital data models to produce parts with any geometry [8,9,10,11,12]. Among AM techniques, the laser powder bed fusion (LPBF) process, as one of the metal additive manufacturing technologies, uses lasers as energy sources to melt or fuse powder materials for layer-by-layer part manufacturing [13]. The LPBF process, also called selective laser melting (SLM), is considered one of the best methods for forming complex metallic components [14,15]. This technology achieves high-performance parts with complex shapes through multi-cycle micro-zone melting, in which the final product performs with good mechanical properties, high surface smoothness, and complex geometries [16,17]. Regarding LPBF with nickel-based superalloy, due to factors such as low thermal conductivity, constant freezing range, and a high thermal expansion coefficient during the building process, severe shrinkage [18], coarse grain, element segregation, high internal stress, and thermal cracking were commonly presented during the solidification process [19], which significantly compromised the performance of the final formed samples. In the LPBF preparation process, rapidly heating and cooling cycles also promote the occurrence of thermal cracks [20,21,22,23,24], such as CM247LC nickel-based superalloy [25,26], IN939 [27], Inconel 738 [28], and Hastelloy X [29]. As a result, the reliability of additive manufacturing is lower than that of traditional manufacturing technologies. Therefore, it is necessary to solve the problem of crack defects and successfully prepare highly densified nickel-based superalloy via LPBF. One of the main strategies to reduce cracks is to optimize the alloy composition with elements such as Co, Zr, Hf, Si, Mn, and B, which were identified as crucial in determining the sensitivity of high-temperature alloy cracking [30]. Some of high crack sensitivity alloys, such as Hastelloy X, CM247LC, and IN738LC, have achieved good forming performance by reducing the content of high-crack-sensitivity elements [31,32,33,34]. 

Against the backdrop, the research and application of GH3230 (also known as Haynes 230) become particularly important. With its excellent high-temperature mechanical properties and oxidation resistance, GH3230 is widely used in various fields such as aerospace, aviation, and power generation. With the advent of additive manufacturing technology, the LPBF process has emerged as a significant method for the manufacturing of the GH3230 alloy. This process facilitates the fabrication of the GH3230 alloy with a relative density of 99.34%. However, it has been observed that the alloy exhibits poor repeatability in the printing process [35]. Studies have explored that the strength and plasticity of the GH3230 alloy dramatically decrease due to the generation of micro-cracks [36,37]. Given the application of this material in high-temperature environments, the implications of micro-cracks become exceptionally severe, which are now recognized as a critical issue in the research and application of the GH3230 alloy. An effective solution to address the problem of micro-cracks in GH3230 alloy lies in compositional optimization. Adjusting the alloy components and refining the fusion process can significantly enhance its printing performance, reduce the occurrence of micro-cracks, and ultimately elevate the application efficacy of the GH3230 alloy [38].

Based on the above research background, this study applied LPBF in situ to alloying technology as the high-throughput additive manufacturing method, using a powder blend of elemental Fe, elemental Ni, elemental Co and pre-alloyed GH3230 powder with different ratios as raw materials, to fabricate as-built samples with varied element content (around the standard ingredients) and diverse laser parameters efficiently. To investigate the effects of different elements and different process ratios on crack behavior quantitively, an algorithm based on the deep learning approach for detecting crack and hole defects was developed. This paper can provide research methods and a theoretical basis for crack control and special component design of nickel-based superalloy.

## 2. Experimental Materials and Methods

### 2.1. Experimental Materials

**Figure 1:**
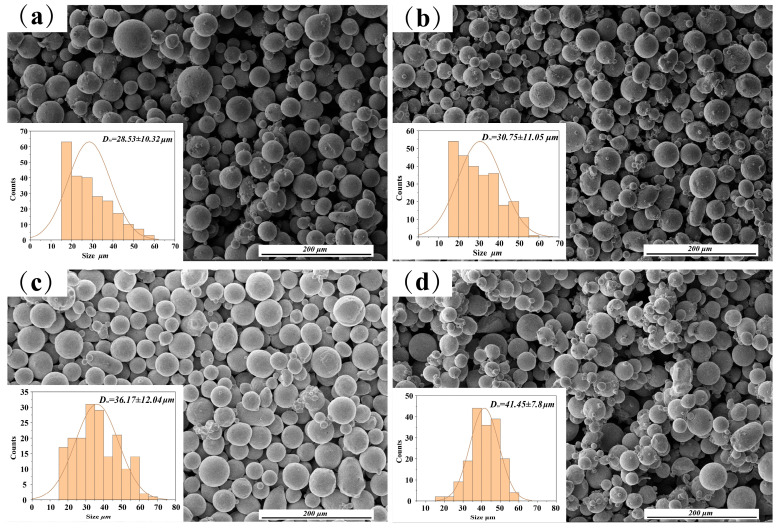
SEM morphologies of powders. (**a**) Ni powder; (**b**) Co powder; (**c**) Fe powder; (**d**) mixed powder.

Experimental raw materials include GH3230 nickel-based superalloy powders and spherical Fe, Co, Ni elemental powders (with purity above 99.5%), which were prepared by atomization process. The so-called “mixed powder” refers to the result of mixing elemental powders with GH230 powder. The morphology of the metal powder observed by scanning electron microscopy (Thermofisher Apreo 2) is shown in Figure 1. The structure exhibits a nearly spherical form of well-dispersed nanocrystals, complemented by a small fraction of satellite spheres. The particle size distribution of the metal powder ranges from 15 μm to 73 μm. The nominal chemical composition of GH3230 powder, provided by CISRI-GAONA, is shown in Table 1. The substrate material for building samples is 316L stainless steel. The subsequent printed samples were characterized using an energy dispersive spectrometer (EDS) detector (Thermo Scientific Apreo 2 SEM), and the results were obtained.

### 2.2. Sample Preparation

**Figure 2:**
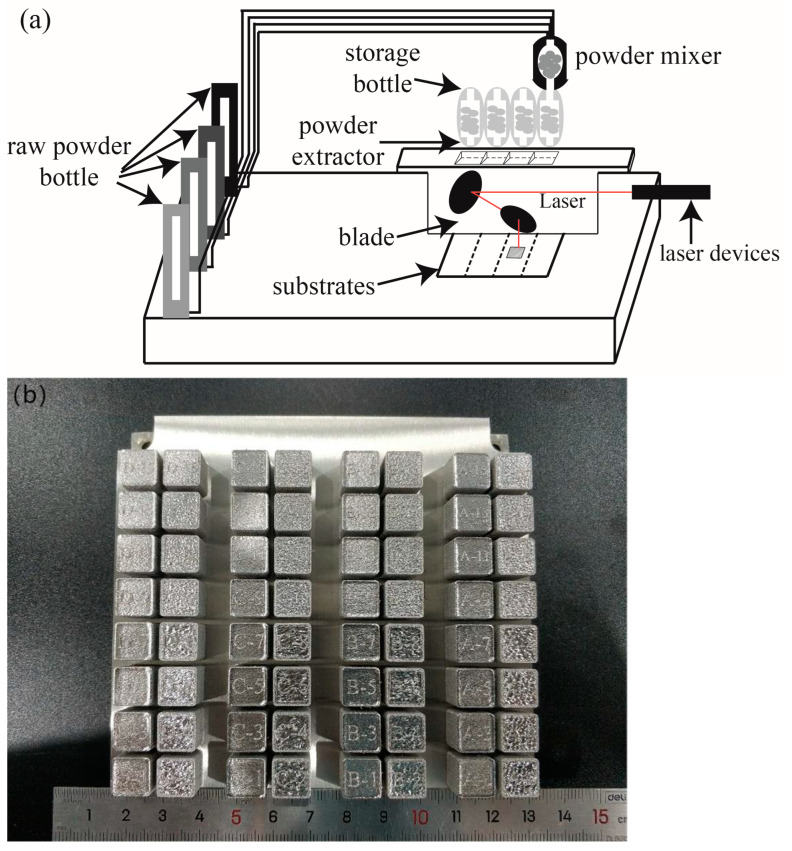
(**a**) Schematic diagram of high-throughput additive manufacturing system (HTAMS) [39] and (**b**) the as-built samples.

Using the self-developed high-throughput additive manufacturing system (HTAMS) [39], the compositional samples of nickel-based superalloy were prepared. The schematic diagram of the equipment structure is shown in Figure 2a; there are 4 powder storage cylinders for real-time quantitative feeding, and the raw powders were transported to the mixing module by Ar2 for high-speed mixing and then sent to different powder distributors. Before the preparation begins, the powder in the raw material powder bottles is transported to the powder mixer according to the set mass ratio through inert gas for mixing. The mixer, connected to a slider, can move left and right. After mixing, the powder is sequentially released into the powder storage bottles below. During the layer-by-layer printing process, the powder collector below the storage bottles extracts and releases the mixed powder in front of the powder spreading blade, which then spreads the mixed powder onto the printing platform for laser-selective melting. The platform is equipped with a 500 W continuous fiber laser with a wavelength of 1064 nm and a dynamic zoom system. The maximum forming size is 120 mm × 120 mm × 150 mm (XYZ). The maximum compositional sample preparation number is 200. Figure 2b shows the samples printed in this experiment. The rightmost column represents the addition of W powder and process variations. We analyzed that, since the maximum power was only 210 W, which is not very high, there were many unmelted W particles in the samples, leading to compositional inhomogeneity. Therefore, we did not discuss the effect of adding W to GH230 in the text.

### 2.3. Experimental Methods

The powders that were dried at 80 °C for 2 h to improve the powder’s flowability were put in a rotary mixer for homogenization with a mixing duration of 3 h at a speed of 50 r/min. Two sets of process parameters were designed in the experiment, which were shown in Table 2. In addition to the different laser powers (180 W and 210 W), other process parameters including scanning strategy were the same. Twenty-two groups of mixing powder ratios are designed in the experiment, in which pre-alloyed GH3230 powder is the base material, and elemental powders are alloying elements. The designed compositions of the powder blends are shown in Table 2. The influence of composition was analyzed by evaluating the cracks and defects of all samples quantitatively.

## 3. Calculation and Modeling

### 3.1. Quantitative Crack Statistics Algorithm

**Figure 3:**
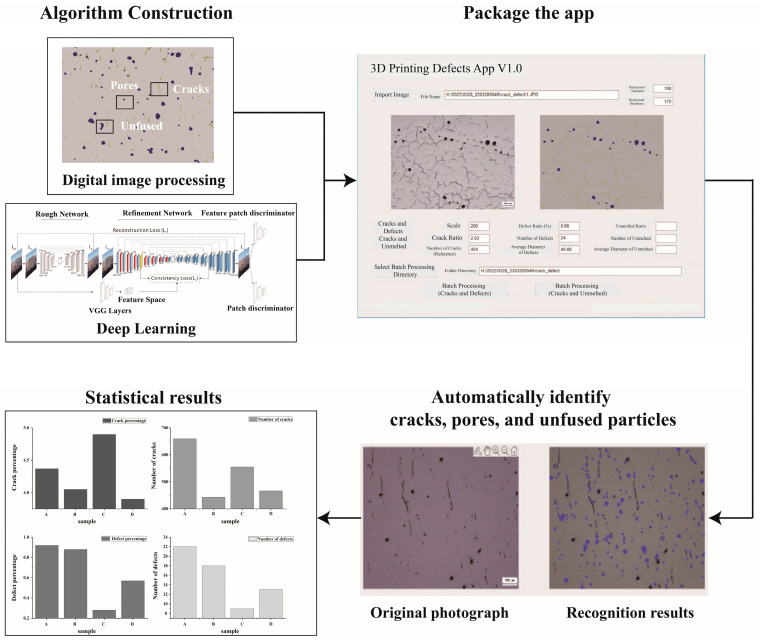
Workflow of defect statistics program based on image recognition method.

This study utilizes a Matlab program [40] developed by our team, which can automatically identify and count the number of cracks, pores, and unmolten particles. The input of the program is based on the optical microscope (OM) photos of the as-built samples; the trained deep learning model constructed based on the decoder-encoder network structure is used to segment and recognize the image to be recognized. For crack defects, the program calculates the percentage of pixels identified as cracks, and for pore and unmolten area defects, the program calculates the percentage of pixels identified as pores or unmelted particles. Each connected region in the recognition result is treated as a separate defect. According to the ruler information read during preprocessing, the pixel area is converted to the real area, and the reduced diameter is calculated. The final quantitative data that can be output include the following: crack volume proportion, hole volume proportion, unmelted particle volume proportion, number of holes and average diameter, and number of unmelted particles and average diameter. Figure 3 illustrates the workflow of the image identification program. In this work, during the identification process of the samples, we have standardized the scale bars and section orientations of each optical micrograph, with approximately 10 images taken for each sample. Additionally, regarding the use of the defect identification program, we aimed to compare the defect quantities across samples rather than discuss the defect counts for each sample individually.

### 3.2. Thermodynamic Calculation

In order to quantitatively evaluate the alloy’s crack sensitivity, this work uses the Thermo-Calc software 2024a [41] with the TCNI12 database for nickel-based superalloy to calculate the curves of phase composition with temperature, curves of solid phase content with temperature, and the freezing range (*FR*) [42]. The *FR* was assessed by calculating the temperature difference between the liquidus and the solidus usually used to predict thermal cracking tendency, that a larger freezing range indicates a greater thermal cracking tendency. Cracks in additive manufacturing of nickel-based superalloy are usually caused by a variety of cracking mechanisms. The solidification cracking index (*SCI*) [43] is one that evaluates the thermal cracking tendency of the alloy based on the square root of the derivative of the final solidification temperature with respect to solid phase content. The work uses the calculated thermodynamic data with Formula (1) to obtain *SCI*. A larger numerical value indicates a greater thermal cracking tendency of the alloy.

(1) $$SCI={\left|\frac{dT}{d(f_{S^{1/2}})}\right|}_{f_{S^{1/2}}\to 1}$$

*T*—Temperature, K; *fs*—Solid phase fraction.

## 4. Results and Analysis

### 4.1. Composition Analysis

**Figure 4:**
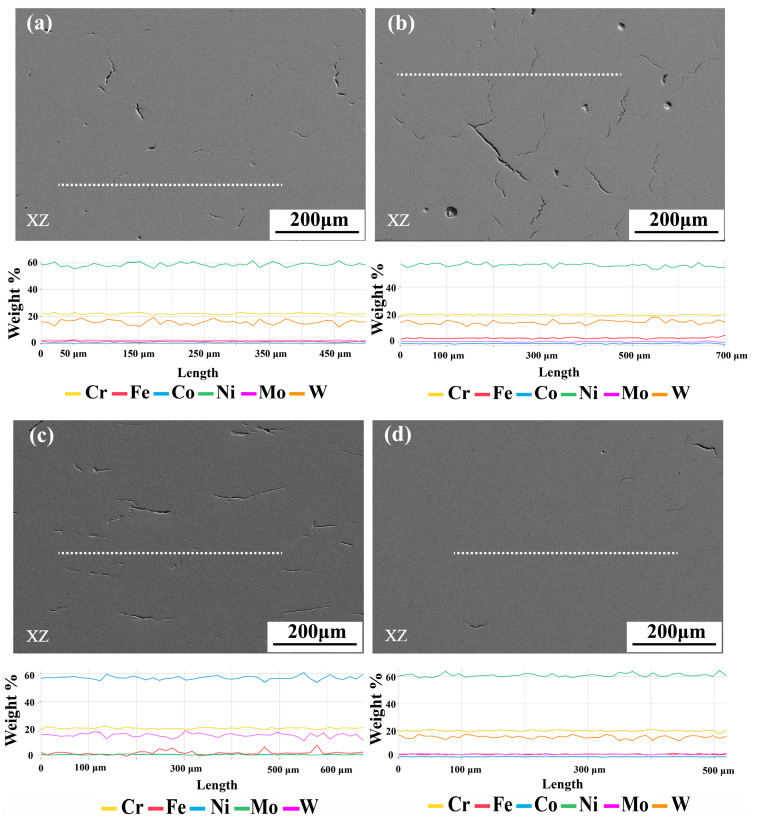
Backscattered photos and line scan curves of different sample with element Cr, Fe, Co, Ni, Mo, and W; (**a**) GH3230 per-alloy; (**b**) sample number 17; (**c**) sample number 18; (**d**) sample number 19.

The characteristic of raw material that using heterogeneous powder could cause uneven element distribution on the macro-scale. The characteristic of LPBF could cause element loss by reason of spattering and evaporation [44,45]. Therefore, the final chemical composition and element distribution of as-built samples are the prerequisites for subsequent experimental analysis. We conducted several experiments in advance on the influence of process parameters on the composition uniformity of the sample, and finally selected the current two sets of process parameters. And we also utilized chemical composition testing by energy-dispersive spectroscopy (EDS). Table 3 shows the absolute error between the designed composition and the tested one. The maximum element composition error is 0.66%. Figure 4 presents the energy backscatter spectroscopy (EBS) and EDS test results for samples with varying Fe content. The EDS line scan composition curves of elements Cr, Fe, Co, Ni, Mo, and W shows minimal fluctuation, indicating good homogeneous alloys, and sufficient interdiffusion of elemental powder in the samples during the LPBF process, with no serious segregation of elements in each sample. The high-throughput additive manufacturing method used in superalloys has high precision in sample composition control.

### 4.2. Influence of Fe and Co Elements on Formability and Crack Behavior 

**Figure 5:**
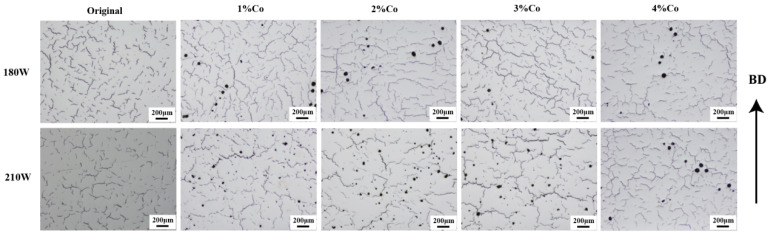
Images of optical microscope for samples with different Co contents in the process at 180 W and 210 W; BD: building direction.

**Figure 6:**
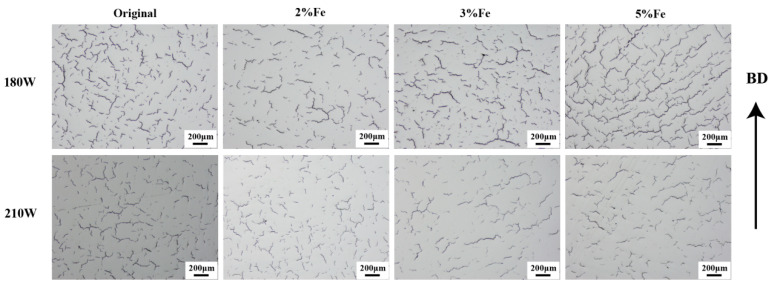
Images of optical microscope for samples with different Fe contents in the process at 180 W and 210 W; BD: building direction.

After verifying the homogeneity of the chemical composition of the samples. this section discusses the impact of the samples with different Fe and Co content on the sample formability during the preparation process. Figure 5 shows the optical microscopy (OM) surface morphology of samples with different Fe content with two sets of laser power, while Figure 6 shows the OM surface morphology of samples with different Co contents at two power levels. It can be observed that samples with the same composition exhibit severe cracking at two different power levels. It has an insignificant impact on cracks when changing the power. The increase in Fe content from 0.55% to 5% results in an insignificant change in the number of cracks, and similarly, an increase in the Co element from 0.005% to 4% leads to severe cracking in the samples.

**Figure 7:**
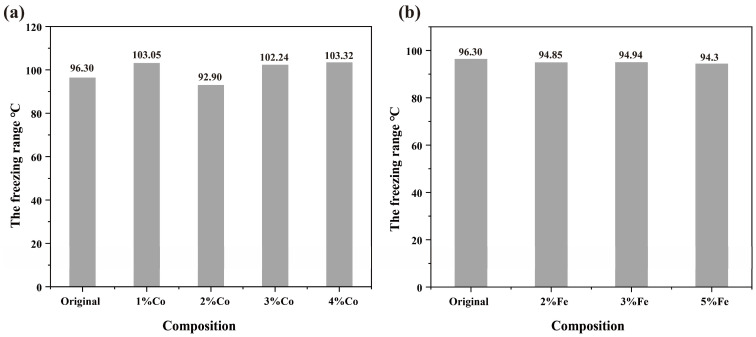
Effect of changes in Co and Fe elements on the freezing range of the alloy in the process at 180 W; (**a**) Co composition involved sample number 9, 10, 11, 12; (**b**) Fe composition involved sample number 17, 18, 19.

**Figure 8:**
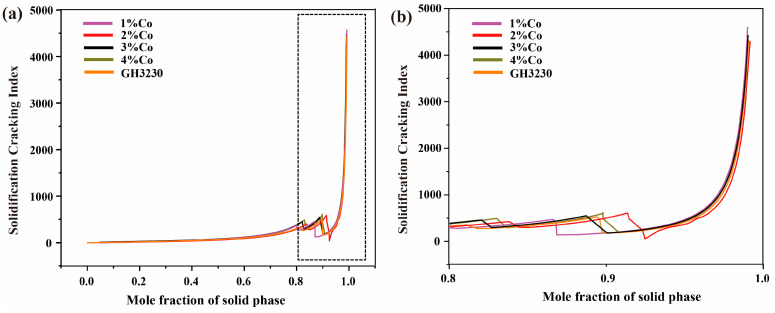
Relationship between solidification cracking index and solid mole fraction of superalloys with different Co compositions involved in sample number 9, 10, 11, 12; (**a**) the entire solidification stage; (**b**) close-up view of the dotted box in (**a**), highlighting the end of the solidification; The dotted box in (**b**) corresponds to the magnified region in (**a**).

**Figure 9:**
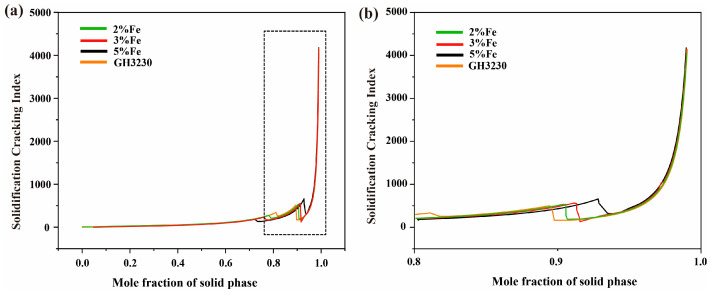
Relationship between solidification cracking index and solid mole fraction of superalloys with different Fe compositions involved in sample number 17, 18, 19; (**a**) the entire solidification stage; (**b**) close-up view of the dotted box in (**a**), highlighting the end of the solidification; The dotted box in (**b**) corresponds to the magnified region in (**a**).

Crack formation is a common problem in the additive manufacturing process. In order to explain the influence of Fe and Co elements on cracks, it is considered that the formation of solidification cracks is controlled by the solidification temperature range and the element segregation behavior at the end of solidification [46]. According to the Scheil equation [47], the wider the solidification temperature range of an alloy, the larger the coexistence interval of the solid and liquid phases, and it will make it easier to produce solidification cracks. The solidification temperature range is related to the alloying compositions. Figure 7 shows the effect of Co and Fe composition changes on the freezing range. It can be observed that Fe and Co alloys with different compositions have significant freezing ranges, with the maximum values being 96.3 °C and 103.3 °C; it can easily lead to cracking, and changes in Fe and Co compositions could not significantly reduce the freezing range. In addition, Kou et al. [42] proposed that the differences in cracking sensitivity at the end of alloy solidification can be described using the solidification cracking index. The freezing range and solidification cracking indices of different elements were calculated. Figure 8 presents the relationship between the solidification cracking index and the solid phase molar fraction (*fs*) for alloys with different Co compositions. From Figure 8a, it can be seen that when *fs* > 0.8, the solidification cracking index of all alloys significantly increases at the end of solidification, indicating a sharp increase in cracking sensitivity. For alloys with a wide solidification temperature range, the phenomenon of insufficient liquid phase shrinkage is more severe [48], resulting in a faster increase in cracking sensitivity. When 0.9 < *fs* < 0.99, there is no significant difference in the solidification cracking index among different alloys, as shown in Figure 8b. Similarly, Figure 9a,b illustrates the relationship between the solidification cracking index and the solid phase molar fraction (*fs*) for alloys with different Fe compositions.

### 4.3. Influence of Ni Element on Formability and Crack Behavior

**Figure 10:**
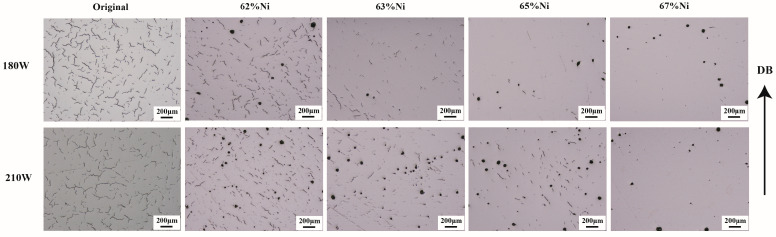
Images of optical microscope for samples with different Ni contents in the process at 180 W and 210 W; BD: building direction.

**Figure 11:**
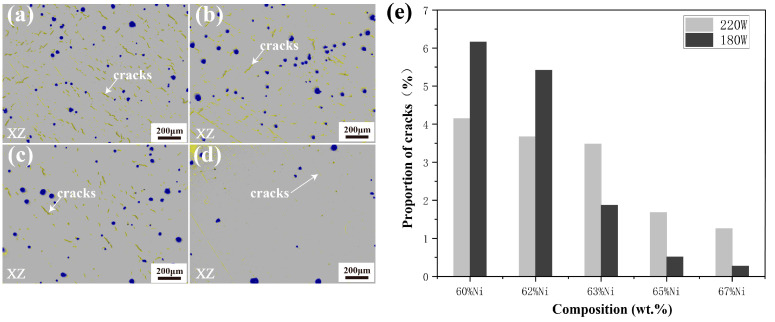
(**a**) Images of crack identification in sample number 1; (**b**) images of crack identification in sample number 2; (**c**) images of crack identification in sample number 3; (**d**) images of crack identification in sample number 4; (**e**) sample number 1, 2, 3, 4 and original alloy crack area proportion statistics.

**Figure 12:**
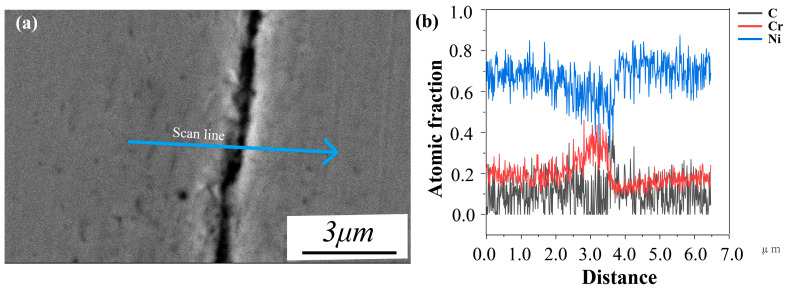
(**a**) Backscattered photo of sample number 1; the blue line is the line scan. (**b**) An EDS line scan profile of line in (**a**) showing the elements distribution of C, Cr, and Ni.

This section studies the effect of the Ni element addition on cracks behavior. As shown in Figure 10, when increasing Ni to 67 wt.% in the process at 180 W and 210 W, the proportion of cracks is relatively decreasing, but when increasing Ni to 63% and 65% in the process at 180 W and 210 W, obvious cracks and porosities appear, and the proportion of cracks is the highest in the original alloy. Figure 11 quantitatively analyzes the crack area fraction in alloys with different Ni contents, showing that the area proportion of cracks decreases with the increase of Ni content. Figure 11a shows the crack identification results of sample number 1, and the yellow line represents cracks and the blue dots represent holes. The alloy with a higher proportion of cracks (sample number 1) is used to study the influence of Ni elements on cracks and their formation mechanism. Firstly, element segregation increasing the tendency of the alloy to crack [32] is considered. Figure 12 shows the distribution of elements at the edge of the crack, and the white dotted line is the line scan of elements C, Cr, Fe, Co, Ni, Mo, and W, and elements such as C, W, Cr, and Mo show obvious segregation. As C is a grain-boundary strengthening element, it tends to combine with metal elements (such as Cr) and exist at grain boundaries in the form of carbides. In order to examine the amount of carbides in vol-% and effects of carbide distribution on cracks, the cracks of sample number 1 are further characterized.

**Figure 13:**
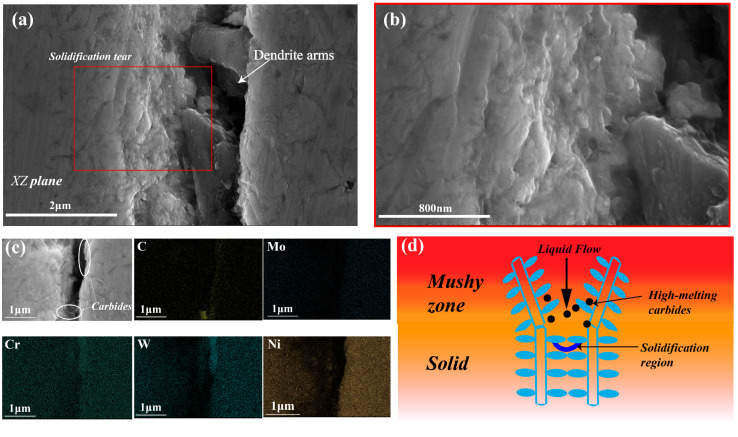
(**a**) Macroscopic morphology photo of solidification cracks of sample number 1. (**b**) The magnified image of the red area in fig (**a**); (**c**) elemental surface distribution of cracks; (**d**) schematic diagram of crack.

Figure 13a shows the microscopic morphology of solidification cracks in sample number 1, and Figure 13b shows the magnified image of the red area in Figure 13a. Figure 13c shows the elemental surface distribution of cracks. Notably, enrichment of CR, C, and W can be observed at the cracks, while high-melting MC carbides often form in the interdendritic region/grain boundary at the beginning of solidification. Due to the pinning effect of carbides, the flow of the liquid phase is hindered, causing the liquid film to be unable to replenish, resulting in shrinkage holes and aggravated stress concentration [49]. At the later stage of solidification, the melt enters a coexistence state of solid and liquid phases, often referred to as the mushy zone [50,51], and the interconnection of secondary dendrite arms of adjacent dendrites hinders liquid backfilling, leading to insufficient liquid for effective compensation [47], as shown in Figure 13d. The concurrent influence of hindered liquid flow and insufficient compensation creates a condition conducive to stress concentration near the interdendritic and grain boundaries. The combination of these two factors leads to stress concentration near the interdendritic/grain boundary, ultimately causing cracking.

**Figure 14:**
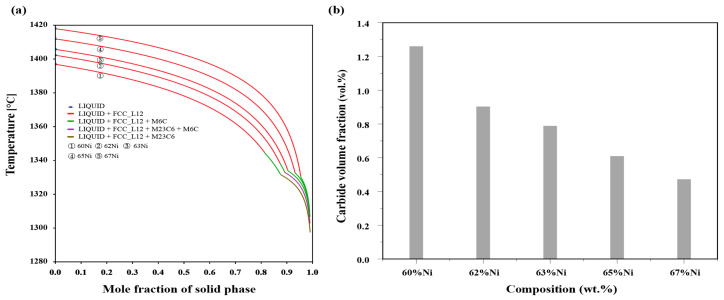
(**a**) Solidification path of sample number 1, 2, 3, 4 and GH3230 pre-alloyed; (**b**) proportion of carbides precipitated during the solidification process of sample number 1, 2, 3, 4 and GH3230 pre-alloyed.

**Figure 15:**
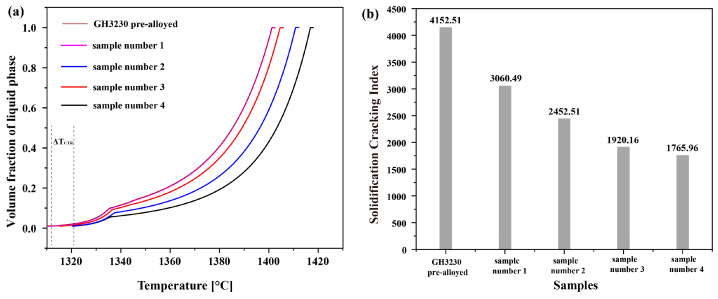
(**a**) Changes in solidification liquid phase volume with different Ni contents of sample number 1, 2, 3, 4 and GH3230 pre-alloyed; (**b**) effect of Ni changes on the solidification cracking index of sample number 1, 2, 3, 4 and GH3230 pre-alloyed at the final stage of solidification.

To further understand the influence of elements on cracks during printing, non-equilibrium solidification curves of samples number 1, 2, 3, 4, and GH3230 pre-alloyed were calculated using Thermo-Calc thermodynamic software, comparing the solidification characteristics of alloys. As shown in Figure 14a, carbides formed in the late stages of the solidification for all alloy samples. In Figure 14b, the carbide content of sample number 1, 2, 3, 4 and GH3230 pre-alloyed was calculated, showing that higher Ni ratios result in smaller volume fractions of carbides, leading to weakened pinning effects and fewer cracks. Additionally, if the alloy only completely solidifies at lower temperatures, the fluidity of the melt deteriorates with decreasing temperature, and the compensatory shrinkage effect also becomes poorer. In the late stages of solidification, when the alloy’s solid phase fraction is high, it is difficult to repair thermal cracks through the liquid phase. If the alloy stays in the critical temperature range [51] (Δ*T_CTR_*) for an extended period, the probability of thermal crack formation increases, and early thermal cracks are more likely to expand into longitudinal cracks, as shown in Figure 15a. Moreover, Figure 15b demonstrates the solidification cracking index of alloys with different Ni contents of samples number 1, 2, 3, 4 and GH3230 pre-alloyed during the late stages of solidification, with the 67% Ni alloy exhibiting lower crack sensitivity and stronger crack resistance. These results are in good agreement with the experimental data.

## 5. Conclusions

In the process of alloy preparation, we focused on observing the influence of different elements on sample quality and crack formation. Through analysis and comparison of these data, the following conclusions are drawn:

1. Samples of GH3230 prepared by high-throughput additive manufacturing based on LPBF in situ alloying technology perform an excellent formability, and demonstrate an accurate element composition; the composition deviation is lower than 0.69 wt.%. However, the adjustment of laser parameters has no significant effect on inter-micro-crack control.
2. Regarding the impact of increased Co and Fe element content on crack defects, our experiments did not yield significant effects in reducing sample cracks. The analysis suggests that Fe and Co alloys have a large freezing range and solidification cracking indices, making them prone to crack formation. Additionally, the fluctuations in Fe content resulted in a mere 2 °C change in *FR*, while variations in Co content led to a relatively modest shift of only 7.02 °C in the *FR*. Variations in Fe and Co content did not significantly reduce the freezing range and solidification cracking index.
3. The addition of Ni elements showed a more significant reduction in cracks, effectively lowering the solidification cracking index. Calculational results showed a significant decrease in the volume fraction of carbides during the solidification stage, weakening the pinning effect of carbides and resulting in fewer cracks. Moreover, the increase in Ni content reduced the alloy’s residence time in the critical temperature range (Δ*T_CTR_*), decreasing the probability of thermal crack formation, and significantly reducing the number of cracks.

## Figures and Tables

**Table 1:** Typical chemical composition of GH3230 alloy (wt.%).

**C**	**Cr**	**B**	**Al**	**Fe**	**Mn**	**Mo**
0.1	21.74	0.0002	0.31	0.55	0.34	2.05
**Ti**	**Co**	**Si**	**La**	**W**	**Ni**	**/**
0.011	<0.005	0.47	<0.001	13.99	Bal.	/

**Table 2:** Sample number, process parameters, and designed composition (wt.%).

Powder Blend	Sample No.	Composition of Base Material	Composition of Elemental Powder	Power/W	Scan Speed mm/s	Layer Thicknessμm	Hatch/m	Spot Diameter/μm
GH3230+Ni	1	96.05	3.95	180	750	25	60	50
	2	93.52	6.48					
	3	88.47	11.53					
	4	83.42	16.58					
	5	96.05	3.95	210				
	6	93.52	6.48					
	7	88.47	11.53					
	8	83.42	16.58					
GH3230+Co	9	99.00	1.00	180	750	25	60	50
	10	98.00	2.00					
	11	97.00	3.00					
	12	96.00	4.00					
	13	99.00	1.00	210				
	14	98.00	2.00					
	15	97.00	3.00					
	16	96.00	4.00					
GH3230+Fe	17	98.00	2.00	180	750	25	60	50
	18	97.00	3.00					
	19	96.00	4.00					
	20	98.00	2.00	210				
	21	97.00	3.00					
	22	96.00	4.00					

**Table 3:** Chemical composition (wt.%) of as-built samples fabricated with laser power 180 W.

Sample No.	1	2	3	4	9	10	11	12	17	18	19
Element	Ni wt.%				Co wt.%				Fe wt.%		
Designed composition	62.00	63.00	65.00	67.00	1.00	2.00	3.00	4.00	2.00	3.00	5.00
Measured composition	62.59	63.40	65.69	67.66	0.98	1.73	2.77	3.92	2.10	2.84	4.45
Deviation	0.59	0.4	0.69	0.66	−0.02	−0.27	−0.23	−0.08	0.1	−0.16	−0.55

## Data Availability

Data are contained within the article.
